# Prevalence of bovine leukemia virus (BLV) infection in the northeast of Iran

**Published:** 2014

**Authors:** Shalaleh Mousavi, Alireza Haghparast, Gholamreza Mohammadi, Seyed-Elias Tabatabaeizadeh

**Affiliations:** 1 *Department of Pathobiology, Faculty of Veterinary Medicine, Ferdowsi University of Mashhad, Mashhad, Iran;*; 2 * Veterinary Medicine, Cell and Molecular Research Group, Institute of Biotechnology, Ferdowsi University of Mashhad, Mashhad, Iran;*; 3 * Department of Clinical Sciences, Faculty of Veterinary Medicine, Ferdowsi University of Mashhad, Mashhad, Iran.*

**Keywords:** Bovine leukemia virus, Dairy cattle, Indirect ELISA, Iran, Prevalence

## Abstract

The purpose of this study was to determine the prevalence of bovine leukemia virus (BLV) in Khorasan Razavi and Khorasan Shomali provinces which are the main provinces located in the northeast of Iran. Total number of 429 blood samples were collected from industrial dairy herds. The samples were categorized based on province, age (2-3, 4-6, and 7-10 years old), calving (≤ 2, 3-5, and > 5) and herd size (≤ 100, 101-250, and > 250) and examined by indirect ELISA. The results of this study showed that 109 (25.4%) out of 429 serum samples were BLV seropositive. The BLV prevalence among cattle of dairy herds of Khorasan Razavi and Khorasan Shomali provinces were 29.8% and 1.5%, respectively. The results showed that the number of seropositive animals was increased significantly with the age (*p *< 0.05). The infection rate in animals 2-3, 4-6 and 7-10 years old were 12.1%, 26.7% and 45.6%, respectively. It was shown that BLV prevalence according to calving ≤ 2, 3-5 and > 5 was 15.5%, 33.0% and 42.9%, respectively, with a significant difference between calving ≤ 2 and > 5 (*p *< 0.001). The prevalence of BLV among herd size of ≤ 100, 101-250 and > 250 was 19.7%, 14.3% and 42.1%, respectively, which was significantly higher in herds with more than 250 cattle (*p *< 0.05). This study revealed that BLV infection in dairy herds of northeast of Iran was influenced by geographical location (province), age, calving and herd size.

## Introduction

Bovine leukemia virus (BLV), a lymphotropic retrovirus structurally related to human T-cell leukemia virus type 1, is the causative agent of enzootic bovine leukosis (EBL), a neoplasm of lymphatic tissue in bovine species.^1^ There are three pathological forms of the disease: asymptomatic course, persistent lymphocytosis (PL) and lymphosarcoma.^2^ The vast majority of infected animals remain healthy with no outward signs of infection and no apparent negative economic effects, but approximately 29.0% of BLV carriers develop PL and less than 5.0% of BLV-infected cattle develop lymphosarcoma.^3^ Naturally, the disease occurs only in cattle but experimentally BLV can easily infect sheep which leads to development of B-cell lymphosarcomas at higher frequencies and after a shorter latent period than cattle.^4^ There is no virus in bloodstream but provirus can be integrated into genome of lymphocytes and tumor cells. Hence, these proviruses are found in cellular fraction of various body fluids which allow transmission via milk, congenital transmission and most commonly, iatrogenic, and horizontal transmission of the disease.^5^^,^^6^ Infection with BLV has a worldwide distribution,^7^ and is listed by World Organization for Animal Health (OIE) as a disease of importance to international trade.^5^ Some European Union (EU) member states and Australia included the disease in the national eradication program and several of which have eradicated the disease.^8^^,^^9^ Because of the lack of vaccine and vaccination, detection of the BLV specific antibody in milk or serum could be a good indicator of the disease and can be exploited as a practical method for disease screening.^5^^,^^10^ Envelop glycoprotein gp51 and viral capsid protein p24 specific antibodies can be found in milk and blood about three weeks after infection.^5^ Enzyme linked immunosorbent assay (ELISA) and agar gel immunodiffusion (AGID) tests are approved by OIE for trading purposes.^5^ ELISA is a rapid method and has a higher sensitivity than AGID and it has been used as a screening test to identify infected dairy herds in regional EBL eradication programs.^9^^,^^11^ Prevalence studies on BLV infection has not been conducted in the northeastern of Iran. The objective of the present study was to estimate the prevalence of BLV in Khorasan Razavi and Khorasan Shomali provinces which are the main provinces located in the northeast of Iran.

## Materials and methods

**Study area. **Khorasan Razavi and Khorasan Shomali provinces are the main provinces located in the northeast of Iran. These provinces have a mountainous area of more than 155000 km^2^. At the lower parts, fertile plants are present which make the suitable condition for development of animal and agricultural husbandry. These provinces are located in north temperature zone and the climate is semi- arid with moderate summers and cold winters.

**Population and sampling. **To date, no comprehensive study regarding the prevalence of the disease in the northeast of Iran has been performed, so the estimated prevalence is considered as 50.0%. Therefore, the sample size required for our study with the desired absolute precision of 95% and an expected prevalence of 50% was calculated using the following formula:^12^

$n=\frac{1.96^{2}P_{\exp}(1-P_{\exp})}{d^{2}}$

where *n* is the required sample size, *P*_exp_ is the expected prevalence, *d* is the desired absolute precision.

The number of the serum samples required by the above formula was 384, however, the ELISA test in the present study was conducted on 429 serum samples. During summer 2009, blood samples (n = 429) were taken from 20 industrial dairy herds of Khorasan Razavi and Khorasan Shomali provinces with total number of 3183 dairy cattle. Seventeen herds from nine cities of Khorasan Razavi and three herds from three cities of Khorasan Shomali were selected according to a random cluster sampling program. The range of dairy cattle in selected herds of Khorasan Razavi and Khorasan Shomali were 493 and 137, respectively. The samples were categorized based on the provinces (Khorasan Razavi and Khorasan Shomali), age (2-3, 4-6, 7-10 years old), calving (≤ 2, 3-5, > 5) and herd size (≤ 100, 101-250, > 250). Blood samples were collected in vacuum tubes (Shandong Weigao Group Medical Polymer Co., Weihai, China) and were put on ice and transferred to laboratory. Samples were centrifuged at 3000 *g* for 15 min at 20 ˚C in laboratory and then the sera were stored at –20 ˚C before analysis by Svanova BLV gp51-Ab ELISA kit (Svanova Biotech, Uppsala, Sweden).

Serological examination. The Svanova BLV gp51-Ab ELISA, an indirect ELISA, was used for detection of antibodies against BLV in serum samples according to manufacturer protocol. The sensitivity (Se) and speciﬁcity (Sp) of the ELISA test were 100 and 99.4%, respectively. Positive, negative and blank controls and the samples were run in parallel. Optical density (OD) values were determined at 450 nm with an ELx800 absorbance microplate reader (BioTek Instruments, Winooski, USA). Before interpretation of the results, all OD values in wells coated with BLV gp51 viral antigen were corrected by subtracting the ODs of negative control from the samples ODs (OD_corrected_ = OD_sample_ – OD_control_). Percent positivity values (PP values) were evaluated. All corrected OD values for the test samples and the negative control were related to the corrected OD values of the positive control as follows:

$\mathrm{PP}=\frac{\mathrm{Test sample or negative control}({\mathrm{OD}}_{\mathrm{corrected}})}{\mathrm{Positive control}({\mathrm{OD}}_{\mathrm{corrected}})}\times 100$

The *PP* value equivalent or greater than 10 were considered positive for BLV infection.

**Statistical analysis. **Statistical analyses were performed using SPSS (Version 12; SPSS Inc., Chicago, USA). The Rogan and Gladen’s correction of apparent prevalence were used for estimation of the true prevalence of the disease which was equated as below. Chi-square statistical method was used for data analysis. Odds ratios and 95 percent confidence intervals were calculated for cattle groups.

## Results

It was demonstrated that 109 (apparent prevalence = 25.4%) out of 429 serum samples were BLV seropositive. However, the true prevalence of BLV antibody-positive was 24.6%. The prevalence was ranged from 0 to 93.3% within the herds. The BLV prevalence among cattle of dairy herds of Khorasan Razavi and Khorasan Shomali provinces was 29.8% and 1.5%, respectively (Table 1).

Herd level prevalence for Khorasan Razavi and Khorasan Shomali was 64.7% and 33.3%, respectively.

The results of our study showed that the number of seropositive animals was increased significantly with age (*p *< 0.05). The infection rate in animals 2-3, 4-6 and 7-10 years old were 12.1%, 26.7% and 45.6%, respectively (Table 1). The estimated odds ratio (OR) for the risk of BLV infection in cattle with 7-10 years old *vs.* cattle with 2-3 and 4-6 years were 6.0 (*p *< 0.05, 95.5% CI = 3.0-11.8) and 2.3 (*p *< 0.05, 95.0% CI = 1.3-3.9), respectively (Table 2).

It was shown that BLV prevalence according to calving ≤ 2, 3-5, and > 5 was 15.5%, 33.0% and 42.9%, respectively (Table 1). There was a significant difference between calving > 5 and ≤ 2 (OR = 4.0, *p *< 0.001, 95.0% CI = 1.8-8.7) and calving 3-5 and ≤ 2 (OR = 2.6, *p *< 0.001, 95.0% CI = 1.6-4.3), (Table 2). We have found that calving ≤ 2 and > 5 had the lowest and the highest prevalence, respectively.

Prevalence of BLV among herd size of ≤ 100, 101-250 and > 250 was 19.7%, 14.3% and 42.1%, respectively (Table 1). The prevalence was significantly higher in herds with more than 250 cattle in comparison with herds with 101-250 cattle (OR = 4.5, *p *< 0.001, 9.0% CI = 1.4-4.4), (Table 2).

**Table 1:** Prevalence of bovine leukemia virus among province, age, calving and herd size groups

**Parameters**		**Positive (%)**	**Negative (%)**	**Total**
**Province**	** Khorasan Razavi **108 (29.8)		254 (70.2)	362
	**Khorasan Shomali **1 (1.5)		66 (98.5)	67
**Age**	**2-3**	17 (12.1)	123 (87.9)	140
	**4-6**	56 (26.7)	154 (73.3)	210
	**7-10**	36 (45.6)	43 (54.4)	79
**Calving**	**≤ 2**	32 (15.5)	174 (84.5)	206
	**3-5**	62 (33.0)	126 (67.0)	188
	**> 5**	15 (42.9)	20 (57.1)	35
**Herd size**	**≤ 100**	39 (19.7)	159 (80.3)	198
	**101-250**	14 (14.3)	84 (85.7)	98
	**> 250**	56 (42.1)	77 (57.9)	133

**Table 2:** Odds ratio values among different cattle groups

**Comparative Parameters**			**OR (95.0% CI) ** ***p*** ** value**	
**7-10 years old**	vs.	2-3 years old	6.05 (3.0-11.8)	*p* < 0.001
		4-6 years old	2.30 (1.3-3.9)	*p* < 0.01
**4-6 years old**	vs.	2-3 years old	2.63 (1.4-4.7)	*p* < 0.001
**Calving 3-5**	vs.	Calving ≤ 2	2.67 (1.6-4.3)	*p* < 0.001
**Calving > 5**	vs.	Calving ≤ 2	4.07 (1.8-8.7)	*p* < 0.001
**Herd size of > 250**	vs.	101-250	4.54 (1.4-4.4)	*p* < 0.001

## Discussion

Previous studies showed prevalence of BLV infection in several cities in Iran.^13^^-^^17^ However, this is the first study of prevalence of BLV infection in northeastern provinces of Iran. Previous studies from other regions of Iran have reported prevalence of 3.0% in cattle in Central province,^13^ 5.7% in Chaharmahal and Bakhtyari province^16^ and 22.3% in Tehran province.^17^ This study that was performed on 429 serum samples taken from dairy herds of Khorasan Razavi and Khorasan Shomali provinces showed a true BLV prevalence of 24.6% (Apparent prevalence = 25.4%).

In this study, the percentage of dairy herds that had at least one BLV-positive cattle was 65.0%. The ELISA test is more sensitive than AGID, however, both tests have high sensitivity and specificity and OIE has introduced them as the standard tests for sero-epidemiological studies.^5^ According to previous surveys and the present study, it could be concluded that since entrance of the high productive breeds to Iran, the prevalence of BLV infection is growing. This increasing BLV prevalence has been a result of lack of planning and management in control and eradication programs at the national levels.

We found that the BLV prevalence among dairy cattle of Khorasan Razavi and Khorasan Shomali provinces was 29.8% and 1.5%, respectively. There are reports of different BLV prevalence from various geographical regions or provinces of a country. Scott *et al*. reported a significantly more BLV-seropositive cows (61.0%) in Manitoba compared with Saskatchewan (37.0%), New Brunswick (29.0%), Nova Scotia (16.0%) and Prince Edward Island (17.0%).^18^ In studies that have been made so far, different results were related to different time of survey, the type of test used and the different geographical regions.^14^^,^^16^ Therefore, different prevalence between these two provinces could be expected based on the different geographical regions and different management practices and risk factors. One of the major reasons in different and significant prevalence between Khorasan Shomali and Khorasan Razavi could be related to low population herds of Khorasan Shomali since we found that herd size was a major risk factor in our present and previous studies.^19^

The results of our study showed that the number of seropositive animals was increased significantly with the age, calving and herd size. An increase of BLV seroprevalence with age has been reported in many studies.^20^^,^^21^

The longer lifespan results in a longer period of BLV exposure, which likely leads to a higher prevalence of BLV infection among dairy cattle.^6^^,^^16^

Our results showed that the BLV prevalence was significantly higher in herds with more than 250 cattle. Intensive dairy production in dairy herds of Khorasan Razavi and Khorasan Shomali provinces is based on a loose housing system which results in an increased physical contact among cattle and could be a primary cause of BLV transmission.^22^ In a recent study, Kobayashi *et al*. showed that a loose housing system was a BLV transmission risk factor.^23^ Therefore, this significantly higher BLV prevalence in dairy herds with more than 250 cattle which are primary located in Khorasan Razavi could be an effect of higher density in herds. In our previous study which was performed on bulk tank samples of dairy herds in Mashhad area, the capital city in Khorasan Razavi province, we found a significant and positive correlation between herd size and bulk tank milk antibody level,^19^ which is consistent with the results of the present study. 

This study showed a prevalence of BLV infection among dairy herds in Khorasan Razavi and Khorasan Shomali. One of the important risk factors which should be considered for control and prevention of the disease is the effect of different management practices among the studied dairy herds. Regarding housing condition, some studies showed that a loose housing system was positively associated with seroprevalence compared with a tied housing system.^22^ Dairy herds of this study used loose housing system which could increase the chances of contact between uninfected and infected cattle and it could be a risk factor that caused BLV prevalence.

As we observed dehorning was a daily practice in herd management of these dairy herds and since dehorning apparatus was not disinfected during dehorning practice so it could be accounted as a risk factor for BLV transmission among calves.^24^ Using common injection needle during vaccination was also a regular procedure in vaccination of the herds. However, reportedly, the quantity of infected lymphocytes passed during common needle injection is not enough to induce BLV infection.^25^ In this study, cows were regularly examined with common sleeves during pregnancy diagnosis, reproductive examination and artificial insemination. Blood contaminated sleeves may transfer the infection between individual cows which could be accounted for BLV transmission in these herds. ^26^^,^^27^

Another possible method of BLV transmission within the herds can be through hematophagous insects,^28^ and due to its presence in the studied herds especially at summer season, we should consider it as an important risk factor associated with the prevalence of infection.

It has been shown that milk and colostrum from infected cattle contain BLV infected cells.^29^ However, all BLV positive cattle do not produce infected milk at all times.^30^ Since colostrum contains BLV antibodies, ingestion of colostrum from infected cows reduces the risk of infection during the weaning period in calves.^31^ However, it has been reported that feeding of infected bulk milk can cause infection in neonatal calves especially from healthy dams.^32^ It was shown that BLV transmission rates in calves by 6-12 months of age can be related to milk born infection at approximately 6.0-16.0% in dairy herds.^33^ In dairy herds of Khorasan Razavi and Khorasan Shomali, after calves get colostrum from their dams, they are fed by pooled milk which can transmit the BLV from infected cattle to calves. This common practice of feeding bulk milk to calves, unprotected by maternal antibody, is likely to be a major factor for the transmission of BLV infection in our study.^27^^,^^34^ Colostrum feeding could be an effective way for reducing the infection in dairy herds. Dairy herds of the present study should be provided by the best conditions for the treatment of pooled colostrum, such as heating and freezing on the herd, and bulk milk from uninfected cows or milk replacement should be fed to calves. Changing or washing sleeves between each rectal palpation should be considered for prevention of infection transmission.

In conclusion, we showed for the first time prevalence of BLV in dairy herds of Khorasan Razavi and Khorasan Shomali provinces of Iran which are the major poles of dairy production in northeast of Iran. In addition, estimated odds ratio revealed a significantly higher risk of BLV infection which was increased by herd size, age and calving. We found the same effect of age and calving on BLV prevalence, therefore, we should consider the effect of calving on BLV prevalence as an effect of higher age and exposure of dams to BLV. Regarding the observed prevalence and economic losses associated with BLV infection, prevention and control programs should be implemented.
